# Autotaxin activity predicts transplant-free survival in primary sclerosing cholangitis

**DOI:** 10.1038/s41598-019-44762-7

**Published:** 2019-06-11

**Authors:** Amandeep K. Dhillon, Andreas E. Kremer, Martin Kummen, Kirsten M. Boberg, Ronald P. Oude Elferink, Tom H. Karlsen, Ulrich Beuers, Mette Vesterhus, Johannes R. Hov

**Affiliations:** 1 Norwegian PSC Research Center, Department of transplantation medicine, Oslo University Hospital Rikshospitalet, Oslo, Norway; 2 Institute of Clinical Medicine, University of Oslo, Oslo, Norway; 3 Research Institute of Internal Medicine, Division of Surgery, Inflammatory diseases and Transplantation, Oslo University hospital Rikshospitalet, Oslo, Norway; 4 Department of Medicine 1, Friedrich-Alexander-University Erlangen-Nürnberg-Erlangen, Erlangen, Germany; 5 Section of Gastroenterology, Department of Transplantation Medicine, Oslo University Hospital Rikshospitalet, Oslo, Norway; 6 Department of Gastroenterology & Hepatology and Tytgat Institute for Liver and Intestinal Research, Academic Medical Center, University of Amsterdam, Amsterdam, Netherlands; 7 Department of Medicine, Haraldsplass Deaconess Hospital, Bergen, Norway; 8 Department of Clinical Science, University of Bergen, Bergen, Norway

**Keywords:** Primary sclerosing cholangitis, Prognostic markers

## Abstract

Autotaxin has been associated with liver disease severity and transplant-free survival. This study aimed to validate autotaxin as a biomarker in two cohorts of Norwegian large-duct PSC patients, one discovery panel (*n* = 165) and one validation panel (*n* = 87). Serum activity of autotaxin was measured in diluted sera by a fluorometric enzymatic assay. Patients reaching an end-point, liver transplantation or death, (discovery panel: *n* = 118 [71.5%]; validation panel: *n* = 35 [40.2%]), showed higher autotaxin activity compared with the other patients, *P* < 0.001 and *P* = 0.004, respectively. Kaplan-Meier survival analyses showed a strong association between increasing autotaxin activity and shorter liver transplant-free survival (discovery panel: *P* < 0.001, validation panel: *P* = 0.001). There was no relationship between autotaxin activity and the presence of inflammatory bowel disease or occurrence of hepatobiliary malignancy. In a multivariable analysis, high autotaxin activity was associated with an increased risk of liver transplantation or death (hazard ratio 2.03 (95% confidence interval 1.21–3.40), *P* < 0.01), independent from Mayo risk score, an in-house enhanced liver fibrosis score and interleukin-8 in serum. In conclusion, increased serum autotaxin activity is associated with reduced liver transplant-free survival independent from Mayo risk score and markers of inflammation and fibrosis.

## Introduction

Primary sclerosing cholangitis (PSC) is a progressive inflammatory disease of the intra- and extrahepatic bile ducts, leading to strictures, cholestasis, cirrhosis and liver failure. There is no effective medical treatment available to retard the progression of PSC. Median time to liver transplantation is 13–21 years^1^. The disease is complicated by cholangiocarcinoma in 10–15% of the patients, which is an important clinical problem with dismal prognosis^2^.

Pruritus is a common symptom in patients with cholestatic liver diseases, but the underlying mechanisms are poorly understood^3^. Autotaxin is an enzyme that hydrolyzes lysophospholipids into lysophosphatidic acid (LPA), which has been identified as a potential mediator of cholestasis-associated pruritus^4^. LPA is unstable and autotaxin activity in serum is used as surrogate marker of LPA levels^5^. Interestingly, autotaxin is overexpressed in some cancers and has been linked to both tumor cell proliferation^6,7^ and fibrosis^8^.

The disease activity in PSC patients shows large variations over the disease course, and there are few tools available to predict prognosis and measure response to treatment for the individual patient. We have previously identified several biomarkers of disease severity and prognosis in PSC reflecting different aspects of the disease process, like inflammation (IL-8)^9^, neutrophil activation (soluble CD14)^10^, fibrosis (enhanced liver fibrosis [ELF] score and pro-C3)^11^ and gut microbial activity^12,13^. Recently, autotaxin activity was described as a marker of severity of liver injury and survival in a study of a single cohort of patients with primary biliary cholangitis (PBC) and PSC^14^. Hence, in the present study we aimed to validate the predictive value of autotaxin activity in two independent Norwegian PSC panels, and explore the role of autotaxin as a potential biomarker for cancer development as well as associations with other novel biomarkers.

## Patients and Methods

### Patient population and data collection

In the discovery panel, 165 patients (74% male) with large-duct PSC were included, while the validation panel comprised 87 patients with large-duct PSC (82% male). Detailed demographic data for both panels can be found in Table 1. The median observation time was 6.58 years (range 0.01–24.36) from time of sampling for the merged panel.

**Table 1:** Demographic information for the two study panels.

	Discovery panel	Validation panel	*P*
	*n* = 165	*n* = 87	
Males, n (%)	122 (74)	71 (82)	
Age at sampling, years, median (range)	40.98 (13.23–75.50)	38.48 (16.33–63.47)	0.866
Age at diagnosis, years, median (range)	34.06 (13.22–70.09)	34.30 (14.48–62.22)	0.821
PSC duration, years, median (range)	1.08 (−0.15–22.39)	0.72 (−0.59–28.95)	0.328
IBD ever, n (%)	137 (83)	68 (78)	0.037
Follow-up, years, median (range)	4.33 (0.01–24.36)	6.77 (0.04–8.35)	0.335
Liver transplantation as end-point, n (%)	86 (52)	27 (31)	
Death as end-point n, (%)	32 (19)	8 (9)	
Mayo risk score, median (range)	0.73 (−2.24–5.26)	0.07 (−2.10–3.28)	0.005
ELF-score, median (range)	11.14 (7.67–14.29)	10.67 (7.86–14.11)	0.004
**Laboratory data**			
ALP, U/L, median (range)	567 (70–3100)	230 (51–1459)	<0.001
ALT, U/L, median (range)	108 (8.0–780)	88 (14–885)	0.107
AST, U/L, median (range)	100 (8.0–1012)	65 (16–1219)	0.014
Albumin g/L, median (range)	37 (15–50)	41 (24–50)	<0.001
Total bilirubin, μmol/L, median (range)	31 (5–464)	20 (4–532)	0.012
Creatinine, μmol/L, median (range)	73 (41–216)	68 (39–90)	<0.001
Platelets 10^9^, median (range)	233 (10–879)	285 (56.7–819)	0.008
**Other information**			
Gall bladder cancer, n (%)	1 (0.6)	0	
Cholangiocarcinoma, n (%)	18 (11)	8 (9)	
Hepatocellular carcinoma, n (%)	2 (1.2)	0	

ALP, alkaline phosphatase; ALT, alanine aminotransferase; AST, aspartate aminotransferase; ATX, autotaxin; ELF, enhanced liver fibrosis; IBD, inflammatory bowel disease; PSC, primary sclerosing cholangitis.

Diagnosis of PSC was based on typical findings on magnetic resonance cholangiography or endoscopic retrograde cholangiography according to established criteria^15^. Patients were recruited at admission to Oslo University Hospital Rikshospitalet in the period from 1992–2006 in the discovery panel and 2008–2011 in the validation panel. Regarding the end-point of liver transplantation or death, patients were followed until 31^st^ of December 2016. Blood samples were included into the Norwegian PSC Research Centre (NoPSC) Biobank (Oslo, Norway). The first cholangiography compatible with PSC defined the time of diagnosis. Duration of disease was defined as the time from diagnosis to serum sampling. Clinical and demographic information was acquired from patient records and research databases, including laboratory data, history of ascites, encephalopathy, variceal bleeding, inflammatory bowel disease, colorectal or hepatobiliary malignancy and medication at the time of serum extraction. Inflammatory bowel disease was diagnosed based on endoscopic and histological findings, according to accepted criteria^16^. Cause of death was extracted from the Cause of Death Registry (Oslo, Norway, reference 16–0230). Revised Mayo risk score was calculated according to the established algorithm^17^, in addition to Model For End-Stage Liver Disease (MELD) score^18^ and Aspartate aminotransferase (AST) to Platelet Ratio Index (APRI) score, all calculated from data at sample time^19^.

### In-house ELF score

Commercial kits were used to analyze serum levels of TIMP-1 and hyaluronic acid (both R&D Systems), and intact N-terminal PIIINP was analyzed by radioimmunoassay (UniQ PIIINP RIA; Orion Diagnostica, Espoo, Finland) in frozen serum samples. Inter- and intra-assay coefficients of variation were <10%. The algorithm used in the ADVIA Centaur XP ELF test, combining TIMP-1, hyaluronic acid, and PIIINP values, was subsequently used to calculate in-house ELF scores (ELF score = 2.278 + 0.851 ln(C_hyaluronic acid_) + 0.751 ln(C_PIIINP_) + 0.934 ln(C_TIMP1_)).

### Autotaxin activity assay

Stored sera, from sample time, thawed up to two times were analyzed for autotaxin activity by a fluorometric enzymatic assay. Autotaxin activity was quantified in (at least 20-fold diluted) sera. Briefly, serum samples were incubated with a buffer containing 500 mmol/L of NaCl, 5 mmol/L of MgCl_2_, 100 mmol/L of Tris (pH = 9.0), and 0.05% Triton X-100 for 60 minutes at 37 °C. Parallel incubations were performed in the presence and absence of 1 mmol/L of LPC (14:0). Subsequently the amount of liberated choline was determined by enzymatic fluorimetry using choline oxidase (2 U/mL), horseradish peroxidase (1.6 U/mL), and homovanillic acid as substrates for peroxidase. After the addition of both enzymes in a buffer (consisting of 20 mmol/L of CaCl_2_, 2 mmol/L of HVA, 50 mmol/L of 3-[N-morpholino] propanesulfonic acid [pH = 8.0], and 0.1% Triton X-100), the increase in fluorescence was monitored at 37 °C using a NOVOstar analyzer (excitation 320 nm and emission 405 nm; BMG Labtech GmbH, Offenburg, Germany). The (endogenous) amount of choline present in the sample without the addition of LPC was subtracted from the amount measured in the presence of LPC. Inter-assay variance was less than 15%, and intra-assay variance below 10%.

### Ethics

The study was carried out in accordance with the Declaration of Helsinki. Written informed consent was obtained from all study participants. The Regional Committee for Medical and Health Research Ethics of South-Eastern Norway approved the study (reference 2011/2572).

### Statistical analyses

Data are presented as median (range) unless stated otherwise, except survival (in years) which is presented as mean (95% CI). When dichotomizing autotaxin levels in high/low categories, the previously published cutoff (>7.5 nmol mL^−1^ min^−1^) defined by Wunsch *et al*. was used^14^. Comparisons were done with Mann-Whitney U tests. Liver transplant-free survival was analyzed using Kaplan-Meier plots, and univariate and multivariate Cox regression. Variables with P < 0.05 in the univariate analysis were included in the final multivariate Cox regression model, except age, AST, bilirubin and albumin as they are included in Mayo risk score. The MELD score was highly right-skewed even after log-transformation and had a high rate of missing data; thus, we used the Mayo risk score in the Cox regression analyses. For correlations, Spearman’s rank correlation test was applied. Statistical analyses were performed using SPSS (version 24; SPSS, Inc., Chicago, IL). P-values < 0.05 were considered statistically significant.

## Results

### Patient characteristics

There were 165 patients in the exploration panel and 87 in the validation panel. 74% and 82% of the patients were male, median age at sampling was 41.0 and 38.5 years, 83% and 78% had concomitant IBD, 52% and 31% underwent liver transplantation and 32% and 8% died during follow-up in the exploration and validation panel, respectively (Table 1). There was one (0.6%) case of gall bladder cancer and 2 cases of hepatocellular carcinoma (1.2%) in the exploration panel, 18 cases (11%) and 8 cases (9%) of cholangiocarcinoma in the exploration and validation panel, respectively.

### High autotaxin activity is associated with poor prognosis in PSC patients

Mean autotaxin activity in the merged cohort (n = 252) was 6.8 nmol mL^−1^ min^−1^ (standard deviation (SD) 3.7), lower in men, mean 6.3 nmol mL^−1^ min^−1^ (SD 3.0) than in women 8.6 nmol mL^−1^ min^−1^ (SD 4.9), *P* < 0.001. The activity was higher than previously reported in both male (2.5 nmol mL^−1^ min^−1^, SD 0.7) and female (3.2 nmol mL^−1^ min^−1^, SD 1.5) healthy controls^20^, *P* < 0.001 for both.

**Figure 1:**
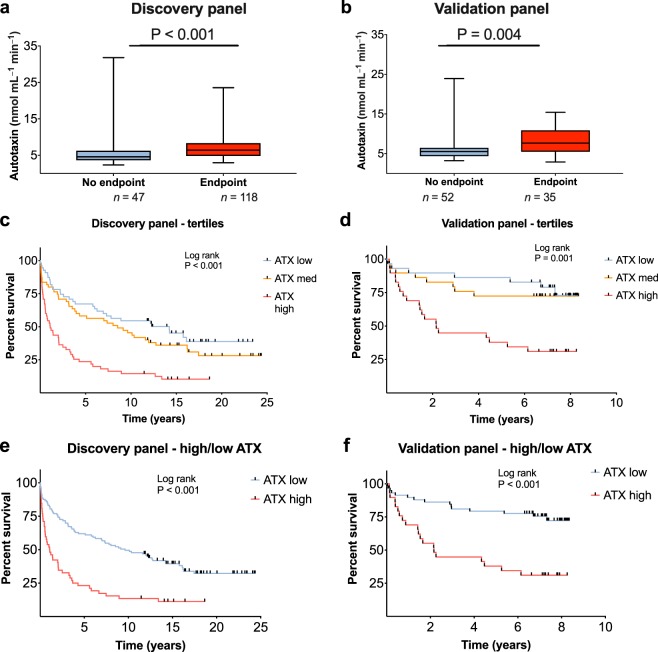
High autotaxin levels are associated with poor prognosis. Autotaxin levels in PSC patients without/with end-point, in (**a**) the discovery panel and (**b**) validation panel. Autotaxin based liver transplant-free survival based on tertiles of autotaxin activity in (**c**) the discovery panel, and (**d**) the validation panel. Poorer prognosis in patients with high autotaxin activity levels (>7.5 nmol mL^−1^ min^−1^ as defined by Wunsch *et al*.^14^), in (**e**) the discovery panel, *P* < 0.001, and (**f**) and validation panel, *P* < 0.001. ATX, autotaxin; PSC, primary sclerosing cholangitis. Data in (**a**) and (**b**) shown as median (min-max).

During follow-up, 118 (71.5%) and 35 (40.2%) patients reached an end-point, liver transplantation or death, in the discovery and validation cohorts, respectively. Patients who reached an end-point during follow-up showed higher ATX activity compared with the other patients in both cohorts (*P* < 0.001 and *P* < 0.004 in the discovery and validation cohort, respectively, Fig. 1a,b).

When stratifying patients according to autotaxin tertiles, there was a strong association between increasing autotaxin activity and shorter liver transplant-free survival in both cohorts (Fig. 1c,d). In a previous study, the optimal cut-off of autotaxin activity to distinguish patients with a higher risk of end-points was 7.5 nmol mL^−1^ min^−1^ ^14^. Repeating the analysis with this cut-off in the present study yielded similar results, with high autotaxin activity concentrations showing reduced liver transplant-free survival compared with the low autotaxin group in both cohorts (Fig. 1e,f**)**, discovery panel: 3.90 years [2.30–5.50] vs. 11.79 [9.95–13.63], respectively, log-rank *P* < 0.001; validation panel: 3.83 years [2.62–5.03] vs. 6.80 [6.07–7.53] years, *P* < 0.001). In a merged panel (*n* = 252) a cut-off of 7.5. nmol mL^−1^ min^−1^ yielded a sensitivity of 35% and specificity of 85% for predicting liver transplantation or death, with corresponding positive predictive value of 81% and negative predictive value of 49%. A dedicated AUROC suggested an optimal cut-off in the present population (using Youden’s index) of 6.4 nmol mL^−1^ min^−1^, with corresponding sensitivity of 54% and specificity of 78%, but with similar positive (79%) and negative (52%) predictive values.

Thirty of the patients with death as endpoint died of liver related causes (75.0%), while nine were registered with non-hepatic causes of death (22.5%, data missing for *n* = 1). Re-doing the above analyses with liver-related deaths only (censoring patients dying from other causes) did not have major impact on the results (data not shown).

### Autotaxin and biochemical characteristics

Autotaxin activity was similar in the discovery and validation panels, mean 6.6 nmol mL^−1^ min^−1^ (SD 3.6) and 7.2 nmol mL^−1^ min^−1^ (SD 3.8), respectively (*P* = 0.28). Despite some baseline differences (Table 1), we therefore merged the discovery and validation panels to increase power when investigating the relationship between clinical and biochemical characteristics.

Significant positive correlations were observed between autotaxin and liver biochemistry (Table 2), with the strongest association observed for bilirubin (rho: 0.376, *P* < 0.001). ALP, AST and INR also correlated with autotaxin (rho = 0.16, 0.30, 0.21, respectively, all *P* < 0.05), as well as C-reactive protein (rho = 0.17, *P* = 0.011), while inverse correlations were noted with creatinine and albumin (rho = −0.323 and −0.404, respectively, both *P* < 0.001).

**Table 2:** Correlations between ATX activity and biochemical markers and clinical risk scores in the merged cohort (*n = *252).

	Spearman’s rho	*P*
INR	0.207	0.007
AST	0.296	<0.001
ALT	0.062	0.344
ALP	0.156	0.021
Creatinine	−0.323	<0.001
Platelets	−0.232	<0.001
Bilirubin	0.376	<0.001
Albumin	−0.404	<0.001
CRP	0.174	0.011
Mayo risk score	0.408	<0.001
MELD	0.233	0.006
APRI	0.355	<0.001

APRI, aspartate aminotransferase to platelet ratio index; AST, aspartate aminotransferase; ALT, alanine aminotransferase; ALP, alkaline phosphatase; CRP, C–reactive protein; INR, international normalized ratio; MELD, model for end-stage liver disease.

**Figure 2:**
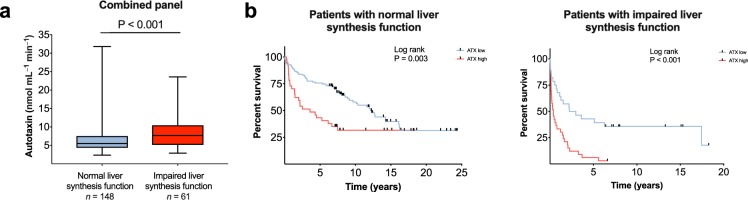
High autotaxin levels are associated with poor prognosis irrespective of liver synthesis function. (**a**) In the combined cohort, patients with impaired liver synthesis function (defined as INR ≥ 1.2 or Normotest < 70) showed higher autotaxin-levels compared to patients with normal liver synthesis function (INR < 1.2 or Normotest ≥ 70). (**b**) Survival curves for patients with normal and impaired liver synthesis function, separated by high/low autotaxin levels (>or ≤7.5 nmol mL^−1^ min^−1^ as defined by Wunsch *et al*.^14^). ATX, autotaxin activity; INR, international normalized ratio. Data on INR and Normotest missing for *n* = 43 in the combined cohort. Data in (**a**) shown as median (max-min).

Patients with impaired liver synthesis function as defined by INR ≥1.2 or Normotest <70 showed increased activity of autotaxin compared with patients with normal synthesis function, combined cohort: 7.69 (2.90–23.56) vs. 5.50 (2.35–31.81), *P* < 0.001 (Fig. 2a). However, autotaxin was a good predictor of liver transplantation or death irrespective of liver synthesis function (Fig. 2b).

### Autotaxin activity and clinical characteristics

In the merged cohort, 30 (11.9%) PSC patients were diagnosed with hepatobiliary malignancy during follow-up. Autotaxin activity was similar in patients who developed cancer during follow-up and those who did not (5.49 [2.90–11.35] vs. 5.81 [2.35–31.81], respectively, *P* = 0.42). There was also no difference in patients with and without IBD (5.89 [2.44–31.81] vs. 6.03 [2.44–22.75], respectively, *P* = 0.90), or between the different subtypes of IBD (data not shown). In a small subgroup n = 32, severity of disease using the cholangiography-based Amsterdam score was evaluated in a previous study^21^. Patients with advanced PSC had numerically higher autotaxin activity than mild PSC (8.9 vs. 7.7), but this was not statistically significant (*P* = 0.51).

Modest significant correlations were found between autotaxin and PSC duration (rho = 0.184, *P* = 0.003), and clinical risk scores; Mayo risk score (rho = 0.408, *P* < 0.001) and MELD score (0.233, P = 0.006), also see Table 2. Data on ursodeoxycholic acid (UDCA) treatment was available for 86 patients from the validation cohort (99%), out of which 35 (41%) were treated at the time of sampling. Patients treated with UDCA had higher autotaxin levels compared to other patients (7.48 [3.83–23.93] vs. 5.50 [2.90–14.25], respectively, *P* = 0.005). However, Mayo risk scores and liver transplant-free survival were not significantly different between patients with and without UDCA treatment (Mayo risk score 0.18 (−2.10–3.28) vs −0.04 (−2.09–3.20), respectively *P* = 0.65, and survival 5.0 (3.8–6.1) vs. 6.4 (5.6–7.3) years, respectively, *P* = 0.055).

### Autotaxin activity and new biomarkers of inflammation and fibrosis in PSC

**Figure 3:**
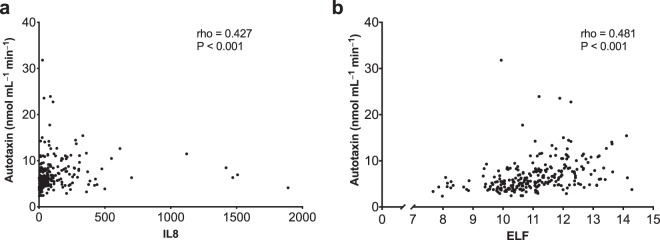
Relationship between autotaxin and markers of inflammation and fibrosis. (**a**) Scatterplot of autotaxin and the inflammation marker IL-8. (**b**) Scatterplot of autotaxin and an in-house ELF score. IL, interleukin; ELF, enhanced liver fibrosis.

**Figure 4:**
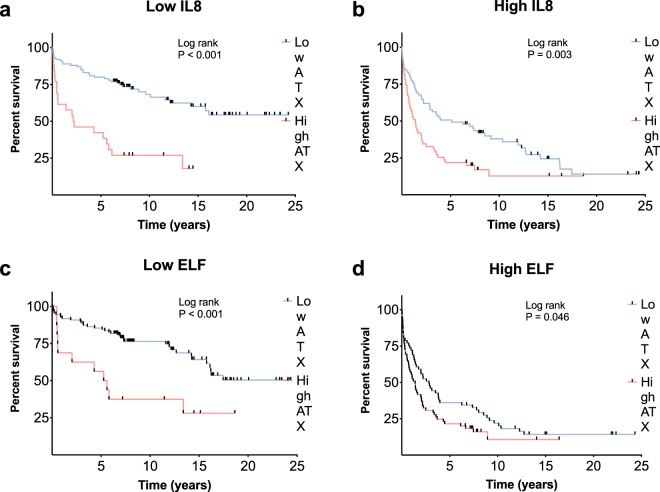
Autotaxin and other established biomarkers in PSC. High autotaxin levels (>7.5 nmol mL^−1^ min^−1^ as defined by Wunsch *et al*.^14^) are associated with poor survival in subgroups with both (**a**) low IL-8 values, and (**b**) high IL-8 values (divided by median). Similar, high autotaxin levels are associated with reduced liver transplant-free survival irrespective of (**c**) low ELF score or (**d**) high ELF-score (divided by median). ATX, autotaxin; ELF, enhanced liver fibrosis; IL, interleukin.

Recent studies have suggested that markers of inflammation (IL-8) and liver fibrosis (ELF test) predict liver transplant-free survival in PSC^11,21,22^. Data on IL-8 and an in-house enhanced liver fibrosis score (ELF-score) were available from the previous studies^21,22^, and both correlated with autotaxin activity **(**Fig. 3**)**. There was a correlation between IL-8 and the in-house ELF-score (rho: 0.656, P < 0.001), autotaxin and IL-8 **(**rho: 0.427, P < 0.001) and autotaxin- and ELF-score (rho: 0.481 P < 0.001). When dividing ELF and IL-8 in high and low by median, and autotaxin in high and low by a cut-off at 7.5 nmol mL^−1^ min^−1^, as defined by Wunsch *et al*.^14^, high levels were associated with reduced liver transplant-free survival for all markers (Fig. 4). Re-doing these analyses with liver-related deaths only, did not influence the results (data not shown).

**Figure 5:**
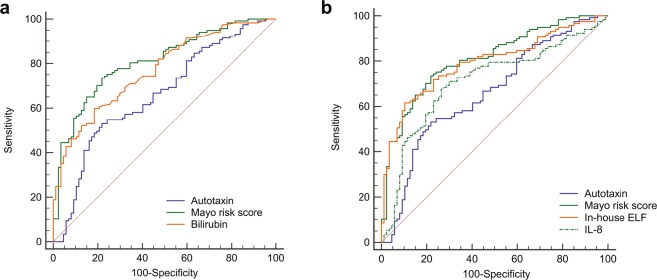
Comparison of autotaxin and other biomarkers in PSC. (**a**) Mayo risk score had a better ability to discriminate patients with and without liver transplantation or death during follow-up (AUC 0.81 vs. 0.66, P < 0.001), as had bilirubin (AUC 0.77 vs. 0.66, P < 0.001), while Mayo risk score and bilirubin were similar (P = 0.07). The same was apparent for the in-house ELF score (AUC 0.79, P < 0.001) but not IL-8 (AUC 0.71, P = 0.25) (panel b). AUC, area under the receiver operating characteristic curve; ELF, enhanced liver fibrosis; IL, interleukin; PSC, primary sclerosing cholangitis.

Notably, Mayo risk score had a better ability to discriminate patients with and without liver transplantation or death during follow-up (AUC 0.81 vs. 0.66, *P* < 0.001, Fig. 5a). The same was apparent for in-house ELF (AUC 0.79, *P* < 0.001), but not IL-8 (AUC 0.71, *P* = 0.25), see Fig. 5b. However, when analyzed using Cox regression, high autotaxin as defined by the validated cut-off 7.5 nmol mL^−1^ min^−1^ was also associated with an increased risk of liver transplantation or death, independent from Mayo risk score, the in-house ELF score and IL-8 (HR 2.03 (95%CI 1.21−3.40), P = 0.007), see Table 3. Adding disease panel (discovery or validation) as covariate, or doing analyses on liver-related deaths only, did not influence the results (data not shown).

**Table 3:** Cox regression analysis in the combined panel (*n* = 252).

	Univariate analysis				Multivariate analysis*		
	*P*	HR	(95% CI)	*n*	*P*	HR	(95% CI)
Autotaxin (high)^†^	<0.001	2.84	(2.05–3.94)	252	0.007	2.03	(1.21–3.40)
Gender (male)	0.216	0.79	(0.55–1.14)	252			
Age at sample	<0.001	1.03	(1.02–1.04)	252			
PSC duration	<0.001	1.06	(1.03–1.08)	252	0.150	1.02	(0.99–1.06)
IBD (yes)	0.632	1.11	(0.72–1.72)	241			
Hepatobiliary cancer (yes)	<0.001	4.04	(2.68–6.09)	252	<0.001	3.25	(1.68–6.25)
Mayo risk score	<0.001	1.76	(1.56–1.97)	204	0.065	1.26	(0.99–1.61)
In-house ELF score	<0.001	2.19	(1.88–2.55)	252	0.066	1.35	(0.98–1.86)
ALP^#^	<0.001	1.42	(1.17–1.73)	219	0.962	1.01	(0.74–1.38)
ALT^#^	0.929	0.99	(0.84–1.17)	233			
AST^#^	<0.001	1.39	(1.15–1.67)	232			
Albumin	<0.001	0.89	(0.87–0.91)	221			
Total bilirubin^#^	<0.001	1.82	(1.58–2.08)	232			
Creatinine	0.801	1.00	(0.98–1.01)	214			
Platelets^#^	<0.001	0.67	(0.54–0.84)	205	0.824	1.04	(0.75–1.44)
Impaired liver synthesis function (yes)^‡^	<0.001	3.09	(2.16–4.42)	209	0.110	1.57	(0.90–2.74)
CRP > 10 (yes)	<0.001	3.89	(2.72–5.57)	213	0.074	1.66	(0.95–2.89)
IL–8^#^	<0.001	1.37	(1.24–1.53)	252	0.934	0.99	(0.80–1.23)

*All variables with P < 0.05 in the univariate analysis were included in the multivariate model, except age, AST, bilirubin and albumin as they are included in the Mayo risk score. Complete data for all variables in the model were available for n = 157 patients in the combined panel. ^†^Autotaxin >7.5 nmol mL^−1^ min^−1^ as defined by Wunsch *et al*.^14^. ^#^Right-skewed distribution, transformed by the natural logarithm before analyses. ^‡^Defined as INR ≥ 1.2 or Normotest < 70. PSC, primary sclerosing cholangitis; IBD, inflammatory bowel disease; ELF, enhanced liver fibrosis; INR, international normalized ratio; AST, aspartate aminotransferase; ALT, alanine aminotransferase; ALP, alkaline phosphatase; CRP, C–reactive protein; IL, interleukin; HR, hazard ratio.

## Discussion

In this cross-sectional study of autotaxin activity in two independent cohorts of PSC patients, we found a strong correlation between increasing autotaxin and shorter liver transplantation free survival. Autotaxin activity correlated with disease severity, as measured by Mayo risk score, but there was no association with IBD or cancer. Autotaxin correlated moderately with new biomarkers in PSC, including IL-8 and ELF-score, but in multivariate analyses high autotaxin was an independent predictor of liver transplant-free survival.

A key observation in the present study is that we show in two independent cohorts of in total 252 large-duct PSC patients that high autotaxin activity is associated with reduced liver transplant-free survival with a moderate HR of 2.0 in multivariable regression, corroborating a previous finding by Wunsch *et al*., who investigated autotaxin in 115 PSC patients^14^. Similar correlations between autotaxin activity and liver biochemistries and clinical parameters like Mayo risk score and MELD score were also seen. This highlights autotaxin activity as a biomarker of potential clinical value, in particular in combination with other markers. Its relationship with disease activity and response to treatment in drug trials should clearly be investigated to define its utility, in line with recent trials investigating markers of fibrosis^23,24^.

The mechanisms causing elevated autotaxin activity in PSC are not clear. Autotaxin has been shown to predict survival, disease stage and cirrhosis also in non-cholestatic conditions^8,25^, while the value of autotaxin as a serum marker of fibrosis varies between phenotypes, being a better predictor in viral hepatitis compared to non-alcoholic fatty liver disease^26^. In other studies of viral hepatitis, autotaxin also correlates with liver stiffness measured with transient elastography^27^. Autotaxin has also been reported to inversely correlate with albumin levels in chronic liver diseases^7,28^, in line with the present data. Mechanistic studies of autotaxin in PSC are scarce. In murine models of fibrosis, increased autotaxin expression in hepatocytes has been observed both after liver injury caused by carbon tetrachloride and after viral infection^29^. Genetic or pharmacological inhibition of autotaxin subsequently reduced liver fibrosis in the model, suggesting a direct link to fibrosis. However, whether hepatocyte expression can account for the increased autotaxin activity observed during cholestasis and cholestatic pruritus in PSC is not known. The negative correlation between autotaxin activity and albumin on the one hand, and positive correlation with INR on the other hand suggest that autotaxin activity is not affected by liver synthesis function. We also make the novel observation that autotaxin activity only moderately correlates with the new PSC biomarkers IL-8 and an in-house ELF, whereas autotaxin activity still independently predicts survival. This suggests that autotaxin is a marker of aspects of the PSC pathogenesis reflecting more than just inflammation or fibrosis. Biomarkers of inflammation, fibrosis and pruritus might independently predict prognosis and underline the potential utility of multi-marker panels to account for the complexity of PSC. This should be investigated in subsequent studies.

Another important observation in the present study is the lack of correlation between autotaxin and biliary tract cancer. This is contradictory to studies describing an emerging role of LPA and autotaxin in malignant diseases^6^, e.g. hematological malignancies^30^ and pancreatic cancer^31^. Autotaxin has been proposed to be an important mediator in cancer cell growth and metastasis^6^. It has also been speculated that it is a link between liver disease and hepatocellular carcinoma^29,32^, but data from human studies are conflicting^33^. Little data is available on cholangiocarcinoma (CCA), and there were no signs of association between autotaxin and biliary cancer in our cohort. One possible explanation is if autotaxin only reflects the presence of tumor without being able to predict later tumor development. Still, the observation contrasts other novel PSC biomarkers, which seem CCA-related. Examples include associations observed between CCA in PSC and elevated ELF^22^, increased soluble CD14^10^ and increased prevalence of IgA anti-GP2^34^, providing further evidence of unique characteristics of autotaxin as biomarker.

A peculiar observation was the increased autotaxin activity observed in PSC patients receiving treatment with UDCA. Importantly, data on UDCA use was only available in the smaller validation cohort (*n* = 87) and an association does not imply causation. In the study by Wunsch *et al*. autotaxin activity was higher in PBC patients who did not respond to UDCA treatment, compared to other patients^14^. It has been hypothesized that the lack of response to UDCA in some PBC patients may indicate a failure to upregulate alk-SMase, another nucleotide pyrophosphatase/phosphodiesterase (NPP) enzyme similar to autotaxin, by UDCA in these patients, leading to increased levels of autotaxin^35^. A more likely hypothesis explaining increased autotaxin activity in UDCA-treated PSC patients in the present study may be that PSC patients in Norway usually receive UDCA only on specific indication, i.e. flares, pruritus or gallstones, and it may be that patients receiving UDCA had more pruritus or even more serious disease. However, it should be noted that measures of disease severity were not convincingly different in patients with and without UDCA (Mayo risk score and liver transplant-free survival).

The strengths of the present study are the large sample size, the inclusion of a validation cohort, and the long follow-up period, although the cross-sectional design makes us unable to make assumptions about cause end effect, which could be improved by repeated sampling during disease activity or after endoscopic intervention. The inclusion from a tertiary care center suggest that the patients have a more severe phenotype than the general population. The limitations also include missing data for some covariates, reducing the number of patients in the multivariate analyzes. We lack data on pruritus, a possible key confounder. There is also a possibility that patients included in the discovery and validation panels could have inherent differences in liver transplantation-free survival due to changes in clinical practice which could introduce a bias, but adding cohort as covariate did not influence the effect of autotaxin. Despite these limitations, it is reasonable to firmly conclude that increased serum autotaxin activity is associated with reduced liver transplant-free survival in PSC patients. This association seems to be independent from Mayo risk score and markers of inflammation and fibrosis, and with no relationship with cholangiocarcinoma development. Autotaxin is therefore an interesting candidate as clinical biomarker, which should be prioritized for follow-up in prospective and interventional studies.

## Data Availability

The dataset used in the present study is available from the corresponding author on reasonable request.
